# Abdominal Hernias, Giant Colon Diverticulum, GIST, Intestinal Pneumatosis, Colon Ischemia, Cold Intussusception, Gallstone Ileus, and Foreign Bodies: Our Experience and Literature Review of Incidental Gastrointestinal MDCT Findings

**DOI:** 10.1155/2017/5716835

**Published:** 2017-05-30

**Authors:** G. Di Grezia, G. Gatta, R. Rella, D. Donatello, G. Falco, R. Grassi, R. Grassi

**Affiliations:** ^1^Radiology Department, Sea Hospital, Naples, Italy; ^2^Radiology Department, University of Campania Luigi Vanvitelli, Naples, Italy; ^3^Breast Surgery Unit, IRCCS Arcispedale Santa Maria Nuova, Reggio Emilia, Italy; ^4^Radiotherapy Department, University of Firenze, Florence, Italy

## Abstract

Incidental gastrointestinal findings are commonly detected on MDCT exams performed for various medical indications. This review describes the radiological MDCT spectrum of appearances already present in the past literature and in today's experience of several gastrointestinal acute conditions such as abdominal hernia, giant colon diverticulum, GIST, intestinal pneumatosis, colon ischemia, cold intussusception, gallstone ileus, and foreign bodies which can require medical and surgical intervention or clinical follow-up. The clinical presentation of this illness is frequently nonspecific: abdominal pain, distension, nausea, fever, rectal bleeding, vomiting, constipation, or a palpable mass, depending on the disease. A proper differential diagnosis is essential in the assessment of treatment and in this case MDCT exam plays a central rule. We wish that this article will familiarize the radiologist in the diagnosis of this kind of incidental MDCT findings for better orientation of the therapy.

## 1. Background

A large number of incidental gastrointestinal findings can be observed during abdominal MDCT exam; they can be divided into benign, indeterminate, and worrisome [1]; even if the most frequent are benign ones, there are several conditions such as abdominal hernias, giant colon diverticulum, GIST, intestinal pneumatosis, colon ischemia, cold intussusception, gallstone ileus, and foreign bodies that represent a clinical medical emergency, and because of that a strait clinical and surgical attention may be needed (Table 1).

## 2. Abdominal Hernias 

Abdominal herniation is a condition characterized by different etiology, types, symptomatology, and treatment. It can be classified into congenital and acquired based on its etiology and into internal and external types [1]; some authors also include diaphragmatic types [2].

Abdominal hernias can be asymptomatic or symptomless and although in many cases the treatment of choice is a surgery intervention, the therapy should be personalized and because of that it is important to take into account complications and recurrences [3].

*Internal hernias* are the protrusion of the intestine through a mesenteric or peritoneal gap within the border of the peritoneal cavity. They can be congenital or acquired; in this second category we can include postinflammatory and traumatic postsurgical herniation, such as after liver transplantation or gastric bypass in bariatric surgery.

Despite the lack of incidence, they can be often misdiagnosed with a 50% mortality in case of strangulation [4]. In relation to the location, they can be distinguished in paraduodenal, through Winslow foramen, intersigmoid, pericecal, transmesenteric, and retroanastomotic; in our personal data 48 out of 84 patients that present to our observation had right or left paraduodenal type (57%) so in line with literature this kind of hernias is the most frequent [5].

### 2.1. MDCT Findings

In left-sided paraduodenal hernia, MDCT can evidence encapsulated bowel loops at duodenojejunal junction between the stomach and pancreas to the left of the ligament of Treitz or between the transverse colon and left adrenal gland; often there is a small bowel obstruction with dilated loops and air-fluid levels; mesenteric vessels can be enlarged, stretched, and displaced; the posterior stomach wall can move anteriorly, the duodenojejunal junction inferomedially, and the transverse colon inferiorly [6].

**Figure 1:**
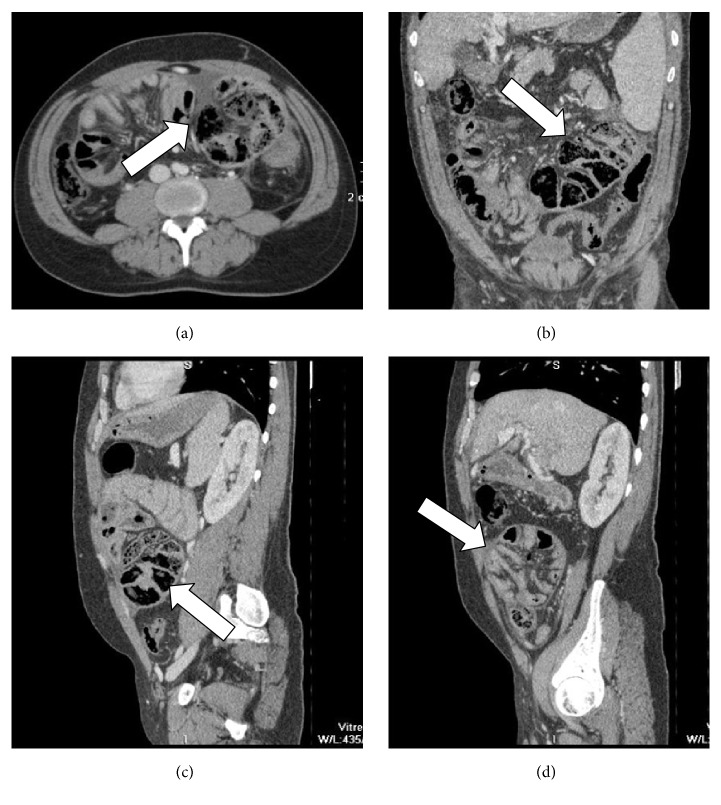
(a) Axial plane, (b) coronal reconstruction, (c, d) sagittal reconstruction of abdominal MDCT exam showing a case of internal hernia, a left side paraduodenal hernia (white arrow).

In right-sided paraduodenal hernia MDCT can show encapsulated loops laterally and inferiorly to the descending duodenum associated with a small bowel nonrotation; superior mesenteric vessels supply the herniated loops [7] (Figure 1).

*External hernias* are the prolapse of intestinal loops through congenital or acquired weakness, defects, or holes of the abdominal or pelvic wall. They include inguinal hernia, umbilical hernia, and femoral hernia. These can be asymptomatic but in some cases a sudden increase in intra-abdominal pressure can lead to a common surgical emergency known as incarcerated hernia.

### 2.2. CT Findings

**Figure 2:**
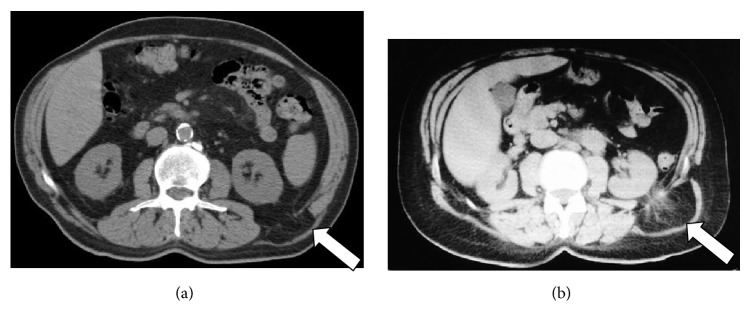
(a, b) MDCT shows external left lumbar hernia (white arrow).

At CT incarcerated hernia appears with bowel dilation and mesangial thickening. A CT scan, followed by oral iodinated contrast administration, is the best method to determine whether the sac content is intestinal and in this case to identify the intestinal type. MDCT scans can help in the detection of bowel strangulation [8] (Figure 2).

Based on our experience in the preoperative assessment, in fact, the identification of a saclike mass or a cluster of dilated small bowel loops located in an abnormal anatomic point is fundamental. At the same time the detection of stretched and displaced mesenteric vascular pedicle and converging vessels at the hernial orifice is vital.

**Figure 3:**
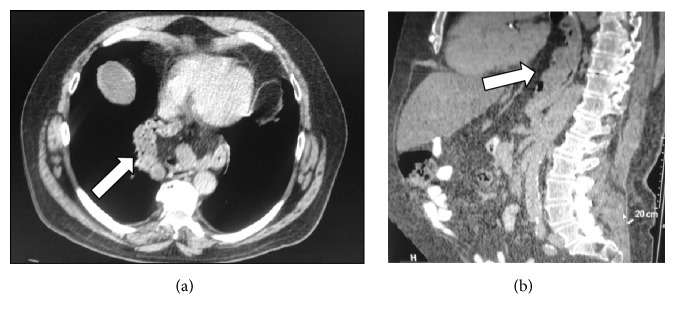
(a) Axial plane and (b) sagittal reconstruction of abdominal MDCT exam showing a case of mixed paradiaphragmatic hernia (white arrow).

*Diaphragmatic hernias (DH)* are defect or hole in the diaphragm that allows herniation of abdominal contents into the chest cavity. DH are defined as congenital or acquired defect in the diaphragm (Figure 3).

Congenital diaphragmatic hernia (CDH) is a rare and severe condition. These defects can range from small subcentimetric defects to complete diaphragmatic agenesis.

Three different types of CDH have been described, which include a posterolateral Bochdalek-type, an anterior Morgagni-type, and a central septum transversum-type or hiatal hernia.

The complications associated with CDH are pulmonary hypoplasia, gastric volvulus, rotational abnormalities, midgut volvulus, hypoplasia of the left ventricle with a left-sided hernia or pleural effusions caused by right-sided involvement, and bilateral renal hypertrophy [9].

### 2.3. US, Chest X-Ray, and CT Findings

In our personal cases (15 patients) during the prenatal period we detected that US has a high sensitivity in the detection of CDH. In the neonatal and infantile periods, a chest radiograph permits an accurate diagnosis. The classic radiographic appearance is a left hemithorax filled with bowel loops with a right-sided mediastinal shifting and no bowel gas is evident in the abdomen [10].

Acquired diaphragmatic hernias can occur for traumatic or iatrogenic causes. Depending on the location and size of the defect, retroperitoneal or intra-abdominal organs and tissues can prolapse into thoracic cavity due to the negative intrathoracic pressure.

CT has been reported to have a sensitivity of 14–82%, with a specificity of 87%. Spiral CT has increased sensitivity, 71–100%, with higher sensitivity on the left than on the right. CT findings indicative of rupture include direct visualization of injury, segmental diaphragm nonvisualization, intrathoracic herniation of viscera, “collar sign,” and peridiaphragmatic active contrast extravasation [11–13].

## 3. Giant Colon Diverticulum

Giant colon diverticulum (GCD) is a rare manifestation of diverticular diseases and is characterized by a large diverticular mass (4 cm in size or larger), in communication with colonic lumen, usually filled with stool and gas. The majority (>90%) arises from the sigmoid colon but it can occur in any part of the colon [14].

Different theories have been proposed to explain the development of GCD but the exact etiology remains unknown. One hypothesis is that it can be caused by a unidirectional ball-valve mechanism through a tiny communicating diverticular neck, which causes air entrapment and gradual enlargement of the diverticulum. Another hypothesis is that GCD is secondary to the action of gas-forming organisms or a true congenital duplication during an anomalous embryologic development [15].

GCD can be divided into three types, based on their histopathological pattern: Type 1: pseudodiverticula (22%); Type 2: inflammatory diverticula (66%); Type 3: true diverticula (12%) [16].

Our patients present with not common symptoms like fever, nausea, vomiting, and rectal bleeding. Other symptoms include constipation and abdominal palpable masses.

The most common complication is peritonitis, caused by the perforation of the GCD, followed by abscess formation, intestinal obstruction, volvulus, and infarction. Rarely, a carcinoma might develop from the diverticular mucosa.

### 3.1. CT Findings

At CT, the diverticulum appears as a cavity filled with gas, fluid, or stool, with a thin regular wall and no contrast enhancement except in the presence of inflammation. The wall may contain calcifications in case of chronic inflammation [17].

The definitive treatment for a GCD is surgery through resection of the involved segment with primary anastomosis.

## 4. Gastrointestinal Stromal Tumors (GIST)

Gastrointestinal stromal tumors (GIST) are uncommon mesenchymal tumors arising from the interstitial cells of Cajal, which express KIT protein-CD117 on immunohistochemistry.

GIST occur not only anywhere along the gastrointestinal tract (GIT), but also in the mesentery, omentum, and retroperitoneum.

The clinical findings are usually site-specific. Lesions in the stomach, small bowel, or colon may present with gastrointestinal bleed in the form of hematemesis, melena, or occult blood in stools; lesions in the esophageal tract presents with dysphagia, but many patients present with vague symptoms, such as nausea, vomiting, abdominal discomfort, weight loss, or early satiety [18].

### 4.1. CT Findings

Although abdominal US is often the primary imaging technique used in the investigation of a patient with abdominal pain or mass we use CT as the modality of choice. It is used to characterize the lesion, to evaluate the extension, and to assess the presence or absence of metastasis. Contrast enhanced CT is also used for monitoring the response to therapy and performing follow-up in case of recurrence [19].

Tumors are usually of varying density and show patchy enhancement after intravenous contrast. Typically the mass has a soft-tissue density with central areas of lower density if necrosis is present and occasionally appears as fluid-fluid levels. Enhancement is typically peripherical and calcification is uncommon. A deep crescent-shaped ulceration demonstrating an internal air-fluid level may be referred to as the Torricelli-Bernoulli sign. This sign can be used to identify ulcerating neoplasms of the GI. Lymph node enlargement is not a feature. Metastases or direct invasion into adjacent organs may be seen in more aggressive lesions.

**Figure 4:**
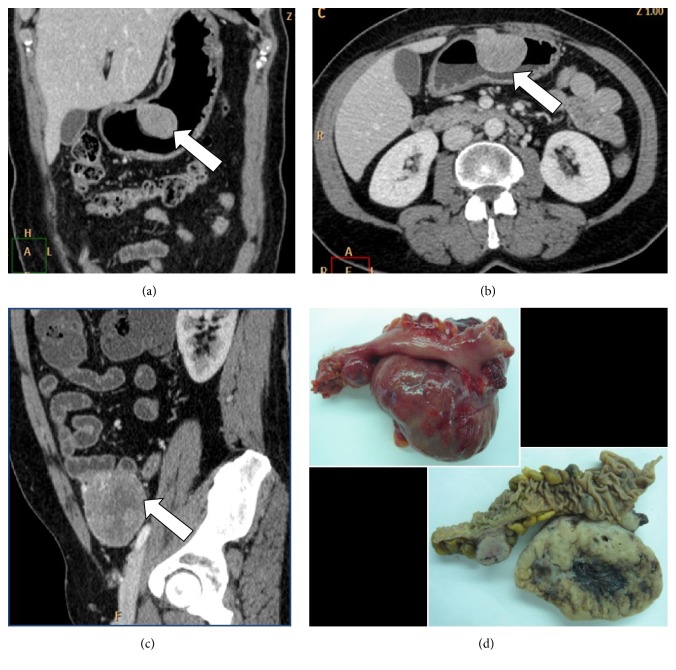
(a) Coronal reconstruction, (b) axial plane, and (c) sagittal reconstruction of abdominal MDCT exam showing cases of gastric GIST (a, b), small bowel neoplasia (c) (white arrow), and postsurgical appearance of the lesions (d).

GIST are positron emission tomography (PET) avid tumors because the receptor tyrosine kinase increases the glucose transport protein signaling. PET is useful in revealing small metastases which would not otherwise be seen on CT [20]. GIST is resistant to a standard chemo- and radiotherapy and has been treated with an advanced molecular targeting therapy or radical surgical excision [21] (Figure 4).

## 5. Intestinal Pneumatosis

Intestinal pneumatosis (IP), also referred to as intestinal emphysema, pneumatosis coli, or pneumatosis cystoides intestinalis, is a rare radiological finding, characterized by gas tracks along the bowel wall, appearing as either linear (submucosal) or rounded cystic collections (subserosal) that occurs in wide spectrum of clinical disorder [22]. The small intestine (42%) is most commonly involved followed by colon (36%), with involvement of both in 22% [23]. PI has been divided into two groups: primary and secondary.

Primary IP (15% of cases) is a benign idiopathic condition in which multiple thin-walled cysts develop in the submucosa or subserosa of the colon. Usually, this form has no associated symptoms, and it is often called pneumatosis cystoides intestinalis [24].

The secondary group (85% of cases) is associated with obstructive and necrotic gastrointestinal disease or with obstructive pulmonary disease.

The pathophysiology of IP has been debated and two main theories have been proposed in the literature. A mechanical theory hypothesizes that gas dissects into the bowel wall from either the intestinal lumen or the lungs via the mediastinum due to some mechanism causing increased pressure or direct trauma. A bacterial theory suggests that gas-forming bacilli enter the submucosa through mucosal rents or increased mucosal permeability and produce gas within the bowel wall.

### 5.1. CT Findings

**Figure 5:**
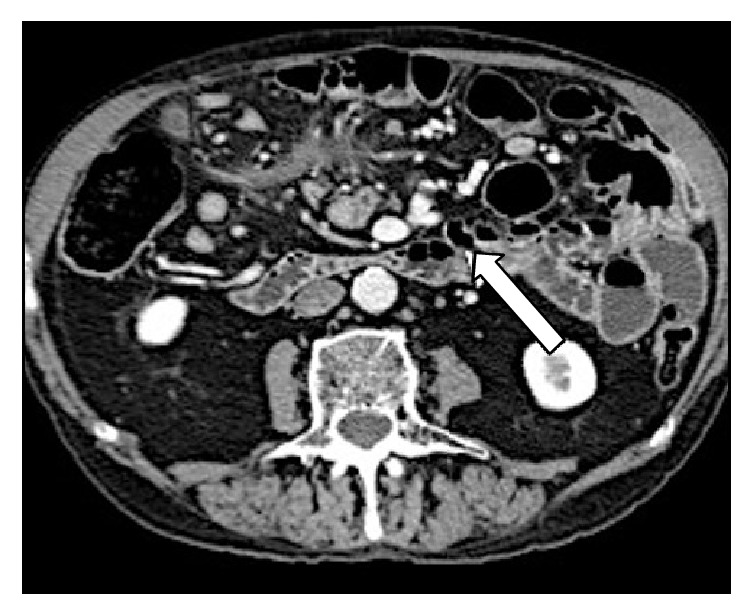
MDCT exam shows small bowel parietal pneumatosis (white arrow).

Computed tomography (CT) is more sensitive than conventional abdominal radiography in the detection of this condition and in the evaluation of extension and complications. The radiological characteristic of IP at CT scan using a lung window is a low-density linear or bubbly pattern or combination of both and gas in the bowel wall. Abdominal CT scanning with or without contrast enhancement can show the morphology, distension, and thickness of bowel loops. CT scans depict additional details, such as morphologic changes, including mural wall thickening, dilatation, abnormal or absent wall enhancement, mesenteric stranding, edema or hemorrhage, vascular engorgement, ascites, and portomesenteric gas, and are helpful in determining the cause of IP (Figure 5).

## 6. Colon Ischemia

Colon ischemia (CI) is a clinical condition that results when blood flow to the colon is reduced to a level insufficient to maintain cellular metabolic function. This process implicates that colonocytes become acidotic and dysfunctional, lose their integrity, and, ultimately, die [25].

The initial and most intense ischemic changes are always in the colonic mucosa. Ischemic change will subsequently extend from the mucosa to the serosa.

The diagnosis of CI is usually established in the presence of symptoms including sudden cramping, mild, and abdominal pain; an urgent desire to defecate; and passage within 24 h of bright red or maroon blood or bloody diarrhea.

CI is often classified according to the underlying cause. Nonocclusive ischemia develops because of low blood pressure or constriction of the vessels feeding the colon; occlusive ischemia indicates that a blood clot or other blockage has cutoff blood flow to the colon.

### 6.1. CT Findings

CT with intravenous and oral contrast is the most helpful in the initial assessment of the patient with abdominal pain to assess the distribution and phase of colitis. It can exclude other causes of abdominal pain, suggest a location and source of ischemia, and identify complications associated with more-advanced disease [26]. The diagnosis of CI can be suggested based on CT findings such as bowel wall thickening (8 mm), thumb-printing, and pericolonic stranding with or without ascites.

**Figure 6:**
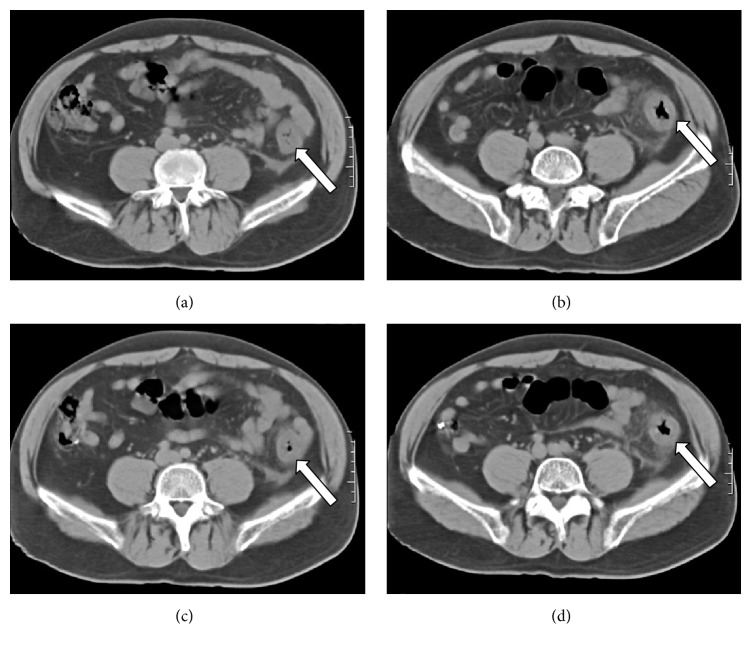
Colon ischemia axial images of MDCT showing bowel wall thickening corresponding to left colon (white arrow).

Most cases of CI resolve spontaneously and do not require specific therapy, and surgical intervention should be considered in the presence of CI accompanied by hypotension, tachycardia, and abdominal pain without rectal bleeding; for pan-colonic CI; and in the presence of gangrene (Figure 6).

## 7. Cold Intussusception

Intussusception is the prolapse of a bowel loop with its mesenteric fold into the lumen of a contiguous segment causing intestinal obstruction. Majority of the intussusceptions are ileocolic, while the remaining are of the ileoileal or the colocolic types [27].

Based on canalization and his consequence, intussusception is classified into three different types: cold intussusception; incomplete and reversible hot intussusception; and complete and irreversible hot intussusception.

Cold intussusception is incidental and asymptomatic with no sign of bowel obstruction such as abdominal pain and obstructive symptoms.

### 7.1. CT Findings

Abdominal CT with mdc with bowel distension by enteroclysis is the most sensitive radiological technique to identify intussusception. CT scans defines the presence (bowel-within-bowel) and intestinal origin of underlying masses, the site and the intestinal tract involved, mesenteric vascular impairment, involvement of perivisceral fat, surrounding tissue, and locoregional lymph nodes.

**Figure 7:**
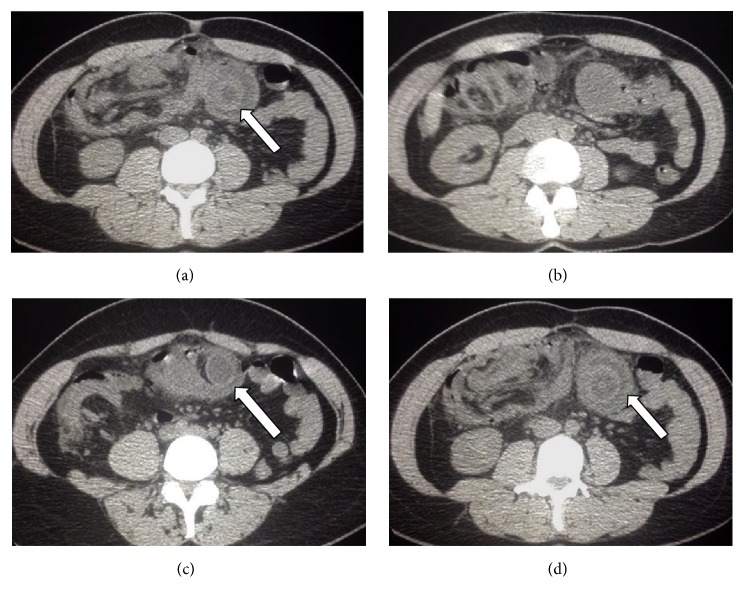
Cold intussusception axial images of MDCT showing the bowel pulled inward into itself (white arrow).

There are three patterns of intussusception that are expression of different stages of the same disease: the target-like pattern (early intussusception with only minimal obstruction and no sign of ischemia); the reniform-pattern (bilobed density with peripheral high attenuation and lower attenuation centrally); and the sausage-shape pattern (alternating areas of low and high attenuation related to the bowel wall, mesenteric fat and fluid, intraluminal fluid, contrast material, or air) [28] (Figure 7).

## 8. Gallstone Ileus

Gallstone ileus is an uncommon cause of a mechanical small bowel obstruction (SBO) due to impaction of one or more large gallstones within the GI tract. Biliary-enteric fistula is the major pathologic mechanism of gallstone ileus. The gallstone enters the GI tract through a fistula between a gangrenous gallbladder and the GI tract. Occasionally a stone may enter the intestine through a fistulous communication between the common bile duct and the GI tract [29]. It is a rare complication of chronic cholecystitis and the most common site of entry by erosion is thought to be to the duodenum.

The clinical manifestations of gallstone ileus are variable and depend on the site of obstruction but are frequently nonspecific with intermittent symptoms of nausea, vomiting, abdominal distension, and abdominal pain.

### 8.1. Abdominal X-Ray and CT Findings

Classically the findings on abdominal radiographs are mechanical bowel obstruction, pneumobilia, and an ectopic gallstone within bowel lumen (Rigler's triad). Contrast enhanced CT evaluation of acute SBO offers prompt and rapid diagnosis of gallstone ileus before operation. The diagnostic criteria of gallstone ileus on CT are as follows:

1. SBO;
2. ectopic gallstone; either rim-calcified; or total-calcified;
3. abnormal gall bladder with complete air collection, presence of air-fluid level, or fluid accumulation with irregular wall [30].

CT also has the capability to estimate size of ectopic gallstone, which renders decision-making in management strategy.

**Figure 8:**
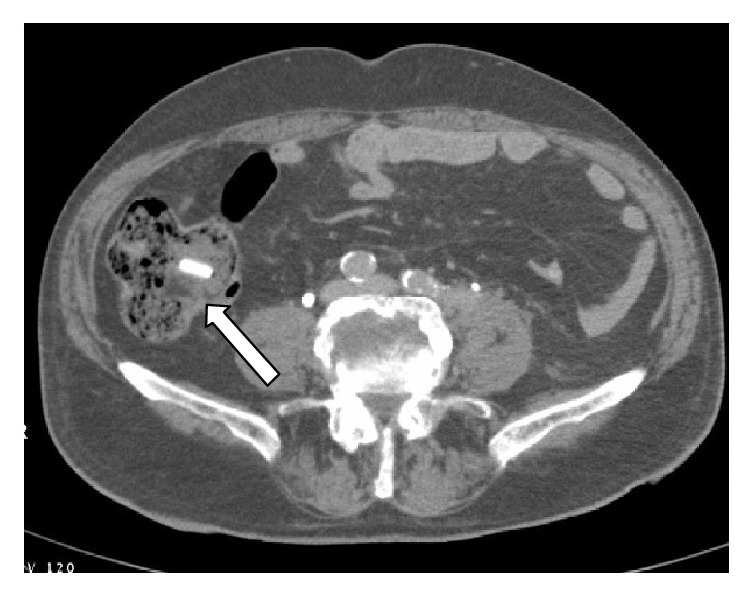
Axial images of MDCT show gallstone ileus (white arrow) in a typical location, the terminal ileum.

The main therapeutic goal is relief of intestinal obstruction by extraction of the offending gallstone [31] (Figure 8).

## 9. Foreign Bodies

Foreign bodies are any object that originates outside of the human body. Foreign bodies may be ingested, inserted into a body cavity, or deposited into the body by a traumatic or iatrogenic injury. The majority of foreign body ingestions occur in pediatric population [32].

Most true foreign bodies can be identified radiographically; however, radiography does not always reliably detect radiolucent foreign bodies, especially fish bones. Even when fish bones are sufficiently radiopaque to be visualized on radiographs, large soft-tissue masses and fluid can obscure the minimal calcium content of the bone, particularly in obese patients. The use of a barium swallow is not recommended because of the risk of aspiration and because coating of the foreign body and esophageal mucosa with contrast interferes with endoscopic visualization.

CT scan is significantly superior to radiography, with a sensitivity from 90% to 100% and a specificity of 93.7% to 100%. With CT, the shape, size, location, and depth of the impacted foreign body and the surrounding tissue can be visualized [33].

**Figure 9:**
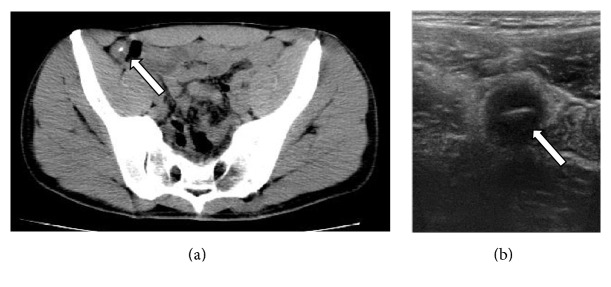
Axial images of MDCT (a) show a foreign body in a small bowel loop (white arrow), also detected in abdominal ultrasonography (b).

The use of IV contrast agent has been long established for the diagnosis of foreign body related complication such as abscess, peritonitis, or fistula formation. Knowledge of these parameters is very important in the management of ingested foreign bodies. The majority of foreign objects pass without intervention and endoscopic removal and surgery are reserved for long, sharp, toxic, or pointed object (Figure 9).

## Figures and Tables

**Table 1:** MDCT mayor criteria in differential diagnosis of incidental gastrointestinal findings.

Abdominal hernias	Internal Left: encapsulated bowel loops at duodenojejunal junction between the stomach and pancreas to the left of the ligament of Treitz or between the transverse colon and left adrenal gland Right: encapsulated loops laterally and inferiorly to the descending duodenum associated with a small-bowel nonrotation; the SMA; and vein drain posteriorly External: bowel dilation and mesangial thickening. A CT scan, followed by oral iodinated contrast administration, is the best method to determine whether the sac content is intestinal and in this case to identify the intestinal type Diaphragmatic: segmental diaphragm nonvisualization, intrathoracic herniation of viscera, “collar sign,” and peridiaphragmatic active contrast extravasation

Giant colon diverticulum	Cavity filled with gas, fluid, or stool, with a thin regular wall and no contrast enhancement except in the presence of inflammation; wall may contain calcifications in case of chronic inflammation

Gastrointestinal stromal tumors (GIST)	Mass with a soft tissue density with central areas of lower density if necrosis is present and occasionally appear as fluid-fluid levels. Torricelli-Bernoulli sign. (PET) avid tumors

Intestinal pneumatosis	Lung window is a low-density linear or bubbly pattern or combination of both and gas in the bowel wall. Abdominal CT scanning with or without contrast enhancement can show the morphology, distension, and thickness of bowel loops

Colon ischemia	Bowel wall thickening (8 mm), thumb-printing, and pericolonic stranding with or without ascites

Cold intussusception	Bowel-within-bowel and intestinal origin of underlying masses, the site and the intestinal tract involved, mesenteric vascular impairment, involvement of perivisceral fat, surrounding tissue, and locoregional lymph nodes

Gallstone ileus	Ectopic gallstone, SBO, abnormal gall bladder with complete air collection, presence of air-fluid level, or fluid accumulation with irregular wall

Foreign bodies	Shape, size, location, and depth of the impacted foreign body and the surrounding tissue can be visualized. IV contrast is not recommended

## Abbreviations

MDCT: Multidetector CT
GIST: Gastrointestinal stromal tumor
DH: Diaphragmatic hernia
CDH: Congenital diaphragmatic hernia
GCD: Giant colon diverticulum
CI: Colon ischemia
IP: Intestinal pneumatosis
SMA: Superior mesenteric artery.
